# Luspatercept in Low-Risk Myelodysplastic Syndrome: A Real-World Single Institution Case Series

**DOI:** 10.1007/s44228-022-00016-4

**Published:** 2022-08-23

**Authors:** Shamis Khan, Sara Taveras Alam, Rosa Torres Ramos, John Etumbani Mbue, Effrosyni Apostolidou, Gustavo A. Rivero, Sarvari Venkata Yellapragada

**Affiliations:** 1 Department of Medicine, Baylor College of Medicine, Houston, TX USA; 2 Department of Hematology and Oncology, Michael E DeBakey VA Medical Center, Houston, TX USA; 3 Section of Hematology and Oncology, Baylor College of Medicine, Houston, TX USA; 4 Baylor College of Medicine, Dan L Duncan Comprehensive Cancer Center, Houston, TX USA

Myelodysplastic syndromes (MDS) are clonal stem-cell disorders caused by inefficient hematopoiesis that result from a complex array genomic and molecular instability. Treatment options for lower risk MDS include supportive care measures such as erythroid stimulating agents (ESAs) and serial transfusions [1]. Luspatercept, a recombinant fusion protein, has recently proven itself as a viable treatment option in patients with low-risk MDS with ringed sideroblasts (RS) and the spliceosome factor 3B1 (SF3B1) mutation [2, 3].

When considering the mechanism of action of luspatercept, it is key to recognize the relevant players in the hematopoietic system that are affected. The target of interest is transforming growth factor-β (TGF-β), an integral member of a superfamily of secreted polypeptide growth factors. TGF-β works in conjunction with the Suppressor of Mothers Against Decapentaplegic (SMAD) circuitry, which functions to convert information from activated TGF-β receptor complexes at the cell surface into transcriptional regulation in the nucleus via multiple potential mechanisms. Concisely, TGF-β signaling causes growth inhibition of hematopoietic stem cells (HSCs) when the SMAD 2/3 complex has partnered with SMAD 4. However, if the SMAD 2/3 complex binds to transcriptional intermediary factor-1 γ (TIF-1 γ), TGF-β will mediate erythroid differentiation.

Luspatercept, consisting of the extracellular domain of activin receptor type IIB fusion protein and the Fc-part of human immunoglobulin G1, exerts its action upon the inhibitory pathway, binding to select TGF-β superfamily ligands that decrease SMAD 2 and SMAD 3 signaling, serving to enhance erythroid maturation and ultimately increase hemoglobin levels [4, 5]. This mechanism has great clinical potential, and this case series will highlight a real-world institutional experience with this medication (Fig. 1).

**Fig. 1:**
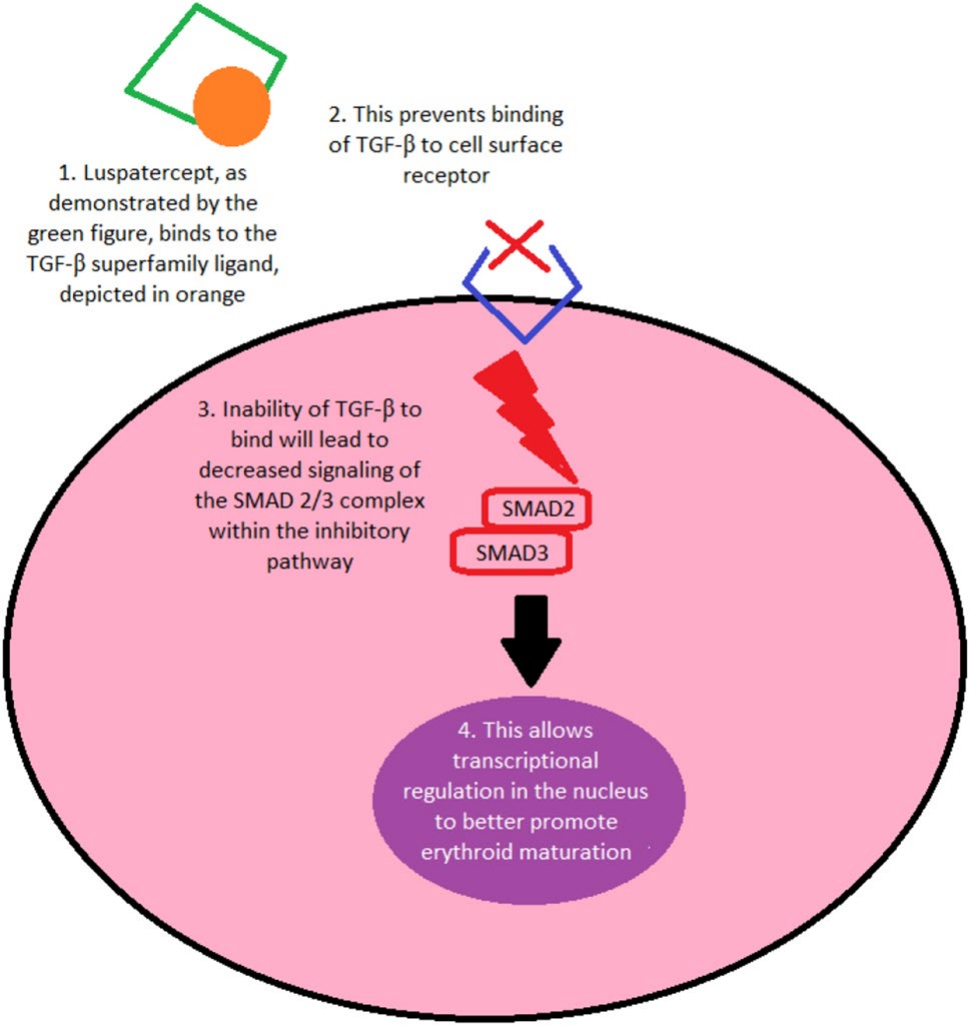
Schematic of the mechanism of action of luspatercept

## Case 1

A 78-year-old male with history of rheumatoid arthritis presented with mild macrocytic anemia and thrombocytopenia. Workup revealed mildly low ferritin and vitamin B12 levels, for which supplementation was started but was ineffective. The biopsy revealed myelodysplastic syndrome with ringed sideroblasts, and cytogenetic analysis of the bone marrow revealed 45 XY karyotype, a chromosome 9;18 translocation, a deletion of the long arm of chromosome 11, and a deletion of the long arm of chromosome 20, suggesting clonal evolution. The Revised International Prognostic Scoring System (IPSS-R) was 0 at the time of diagnosis, indicating very low risk, at that point in time. He was started on darbepoetin alfa, but his hemoglobin decreased further. Workup revealed an SF3B1 mutation and luspatercept was started at 1 mg/kg every three weeks. His haemoglobin demonstrated a 3 g/dL rise within 4 months, dropped during a lengthy hospitalization that led to a treatment holiday, but rose again when luspatercept was resumed. His quality of life improved significantly, but he was noted to have arthralgias of the hips and right shoulder for which the dose was appropriately reduced.

## Case 2

A 73-year-old female with history of thalassemia trait, hypertension, and asthma seen in 2019 for transfusion dependent anemia. After a bone marrow biopsy revealed MDS with RS, ESAs were also ineffective. Chromosomal analysis showed normal karyotype. The IPSS-R score was 2.5, indicating low risk. Further genomic analysis revealed the patient was positive for the SF3B1 and U2AF21 mutations. By July 2020, luspatercept was initiated at 1 mg/kg approximately every month, and her hemoglobin rose from less than 8 to greater than 11 g/dL after 2 doses. Side effects included headaches and musculoskeletal pains for which the dose and frequency of the drug were tapered down to promote greater tolerance.

## Case 3

An 89-year-old male with several comorbidities presented from his nursing home for symptomatic anemia with hemoglobin of 6. A recent bone marrow biopsy revealed MDS with RS, and lab studies demonstrated a vitamin B12 deficiency. His IPSS-R score was 1, indicating very low risk. Transfusions, ESAs, and B12 supplementation did not improve his anemia. After genomic analysis revealed an SF3B1 mutated variant (40%), he was started on luspatercept at 1 mg/kg every three weeks. Since then, his hemoglobin increased from 6.8 to 8.9 g/dL in a span of just 6 weeks and remained in the 8–10 range with transfusion independence. While he tolerated the medication well, he also reported joint pains. A few months later, he was admitted for presumed septic shock, transitioned to hospice, and passed away.

## Discussion

**Fig. 2:**
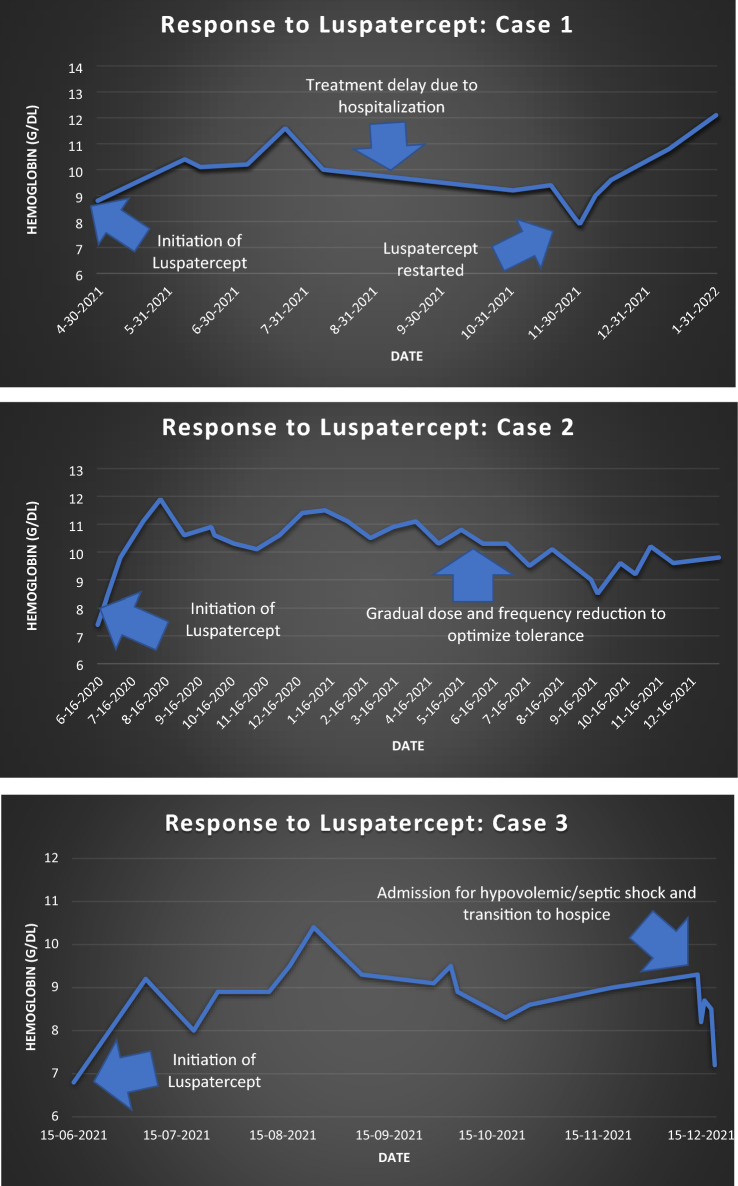
Graphical representations of hemoglobin response to luspatercept

Several themes are present throughout each of the three above cases. Most importantly, each case demonstrates the efficacy of luspatercept in the setting of various classes of MDS with RS and the SF3B1 mutation. In each scenario, ESAs were attempted initially. In patients with lower-risk MDS, studies have shown consistent improvement in erythroid response rates in 45–73% of ESA-naïve patients and 25–75% of patients with prior ESA exposure. In the United States, ESAs are first-line agents in patients who do not have the 5q chromosomal deletion and, in Europe, they serve as first-line treatment in both del(5q) and non-del(5q) MDS [6]. Of note, ESAs have been noted to have a shorter median duration of response in patients with MDS with RS as compared to those without [7]. After demonstrating insufficient response to ESAs, luspatercept was selected and demonstrated hematological efficacy in each case as demonstrated by the Fig. 2.

A second consistent finding amongst the cases was the workup revealing SF3B1 mutations. Roughly half of all MDS patients carry somatic mutations in spliceosome genes, with the SF3B1 gene mutation being the most common one. Given its prevalence, evidence has supported SF3B1-mutant MDS being its own subtype within the family of MDS, as discovery of the mutation has important clinical implication in risk stratification and therapeutic decision-making. Interestingly, the Medalist Trial demonstrated that > 90% of the 37.9% of patients who achieved the primary endpoint had the SF3B1 mutation. This highlights a promising notion that luspatercept is a proven option in the setting of this mutation [8].

Lastly, a common element to each case was the adverse effects related to luspatercept use. In the patients above, the most troublesome effect was bone pain and arthralgias, so much so that the doses of luspatercept were needed to be titrated or suspended in some cases. The trials above indicated that adverse events were common, but seldom led to discontinuation of treatment. Additionally, the incidence of adverse effects in previous studies were noted to decrease over time in luspatercept-treated patients [2]. Thus, while results were promising in the trials, efforts need to be made to improve tolerability in real-world clinical settings.

## Conclusion

In conclusion, luspatercept has proven itself to be a novel therapeutic agent in patients with MDS. More specifically, the patients in this case series were all in the low risk MDS category with symptomatic anemia refractory to ESAs and blood transfusions. In each case, notable positive findings included the presence of RS on morphology and the SF3B1 mutation. Thus, these appear to be key predictive markers that demonstrate the potential for luspatercept to be a therapeutic intervention. While adverse events are not life-threatening, they can affect tolerability, a key point that will need to be addressed as luspatercept becomes more commonplace in the setting of MDS and other disorders that affect hematopoiesis.

## Data Availability

Data sharing not applicable to this article as no datasets were generated or analyzed during the current manuscript.
